# ¿Quién es más susceptible a la información errónea en línea sobre la salud?^*^

**DOI:** 10.26633/RPSP.2021.53

**Published:** 2021-05-12

**Authors:** Laura D. Scherer, Gordon Pennycook

**Affiliations:** 1 Universidad de Colorado Denver Estados Unidos de América Universidad de Colorado, Denver, Estados Unidos de América.; 2 Universidad de Regina Regina Canadá Universidad de Regina, Regina, Canadá.

Si bien cualquier persona podría caer en el engaño de la información falsa, la información errónea en línea no afecta a todos por igual. Algunas personas son más propensas a creer en la información errónea que otras, y actúan como vectores al retransmitirla en los medios sociales. Para combatir la información errónea de forma efectiva en los medios sociales, es crucial entender los factores subyacentes que llevan a ciertas personas a creer y transmitir contenido falso y engañoso en línea. Esta cuestión ha sido abordada por un cuerpo de investigación cada vez mayor, que busca comprender quién es más susceptible a la información errónea en línea y en qué circunstancias. La bibliografía académica sobre la información errónea en materia de salud puede guiar la investigación y las intervenciones futuras. En este artículo se proporciona un panorama general de lo que sabemos acerca de quién es más susceptible a este tipo de información y lo que queda por aprender.

## Perspectivas teóricas

Una perspectiva dominante, conocida como la hipótesis del déficit, es que las personas que creen en la información errónea no tienen un nivel de conocimientos o educación suficiente para discriminar entre información verdadera y falsa. Los investigadores suelen centrarse en la “alfabetización” sobre la salud, pero hay déficits en otros tipos de conocimientos relevantes, como la competencia digital, los medios de comunicación o la cultura científica. Brashier y Schacter argumentan que la razón por la que las personas mayores retransmiten noticias falsas en los medios sociales con más frecuencia que los adultos jóvenes no es el deterioro cognitivo, sino su bajo nivel de competencia digital. Las personas mayores tienen menos facilidad para detectar fuentes de noticias en línea confiables, distinguir entre contenido publicitario o editorial y descubrir fotografías manipuladas (1). En consecuencia, algunas intervenciones han tratado de mitigar la susceptibilidad a la información errónea mejorando el nivel de competencia digital (y las habilidades relacionadas). Por ejemplo, Guess et al. informaron recientemente que una breve intervención de desarrollo de competencias en medios digitales mejoró la detección de titulares de noticias falsas tanto en los Estados Unidos como en la India (2).

Otra teoría es que las personas tienden a ser susceptibles a la información errónea que concuerda con sus creencias o forma de ver el mundo ya establecidas (3). Un cuerpo de investigación notable ha demostrado que las personas tienden a creer información que confirma sus creencias preexistentes (3). Sin embargo, investigaciones recientes han encontrado que la influencia de las actitudes preexistentes puede ser menor de lo que se pensaba. En un estudio en concreto, las personas con un estilo cognitivo más reflexivo, medido por la prueba de reflexión cognitiva, eran más capaces de discernir entre noticias verdaderas y falsas que las personas con un estilo cognitivo más intuitivo (4). Además, lo importante es que este resultado se daba independientemente de si los titulares de las noticias coincidían con la ideología política de los participantes. La tendencia de una persona a hacer mayor uso de un pensamiento reflexivo también se relaciona con su capacidad para detectar información errónea sobre la COVID-19 (5).

Otros trabajos de investigación encontraron que aquellas personas con menor capacidad para discernir entre información verdadera y falsa tienden a sobrestimar sus propios conocimientos y a ser receptivos a frases “seudoprofundas” (los sujetos de estudio califican como profundas frases aleatorias llenas de palabras de moda, pero carentes de significado) (6). Al evaluar todos estos hallazgos en conjunto, los expertos han especulado que la receptividad a la información errónea está relacionada con una actitud más reflexiva y abierta de miras (6). Es decir, las personas más susceptibles a la información errónea ni siquiera se plantean que el contenido pueda ser inexacto, cualquiera sea su ideología política o de sus creencias.

De manera similar, un estudio reciente mostró que un pequeño recordatorio para que las personas reflexionen sobre la veracidad de los titulares es suficiente para aumentar la calidad de las noticias sobre la COVID-19 que estarían dispuestas a retransmitir en las redes sociales (5).

Un experimento realizado en Twitter con una metodología similar también ha producido resultados prometedores (7). Los resultados respaldan la idea de que las personas creen en la información errónea porque no reflexionan sobre la exactitud del contenido que encuentran en las redes sociales, en lugar de por motivaciones políticas o por simple confusión sobre lo que es y no es cierto.

En resumen, hay tres perspectivas teóricas dominantes (no necesariamente excluyentes) que abordan por qué ciertas personas son más susceptibles a la información errónea en línea: 1) están confundidas sobre lo que es verdadero y falso, lo cual indica que el conocimiento y el nivel de alfabetización son un factor central; 2) tienen creencias o motivaciones ideológicas muy arraigadas que conducen a un razonamiento motivado y, por lo tanto, al deseo de creer y retransmitir información errónea acorde, y 3) no reflexionan lo suficiente sobre la veracidad o exactitud del contenido de las noticias encontradas en los medios sociales.

## Preguntas para futuras investigaciones

Por supuesto, otras características individuales no incluidas en las perspectivas teóricas presentadas pueden ser especialmente relevantes a la hora de aceptar información errónea sobre salud. Un factor importante es la confianza en los expertos en salud y las ciencias de la salud. La confianza se articula en varias dimensiones, y una persona puede mostrar distintos niveles de confianza o desconfianza en los médicos, la ciencia médica, los científicos y los sistemas de atención médica. Cada tipo de desconfianza puede hacer que una persona sea más susceptible a la información errónea sobre salud. Se necesita más investigación sobre las diferentes dimensiones de la confianza y sus implicaciones a la hora de creer en la información errónea. Otras características individuales que pueden influir sobre la susceptibilidad a información errónea y que no se han estudiado de manera adecuada incluyen rasgos como la necesidad de autonomía y la actitud hacia la medicina. Por ejemplo, recientemente se encontró una correlación firme entre una actitud medicalizadora (es decir, la tendencia a querer una atención de salud con un enfoque activo y agresivo) y la susceptibilidad a la información errónea sobre la COVID-19. Este resultado justifica una mayor investigación y explicación (5).

Una pregunta clave que aún no ha sido respondida es si la susceptibilidad a la información errónea es un rasgo generalizado o depende del contexto. Las personas que creen la información errónea en el campo de la política pueden ser las mismas que creen la información errónea en el campo de la salud (*5*). Ahora bien, puede haber importantes diferencias entre las personas que creen uno u otro tipo de información errónea, y este tema no ha sido investigado de manera sistemática. De hecho, la información errónea sobre salud abarca muchos temas diferentes, y no está claro si las personas que creen la información errónea sobre un tema de salud en particular, como las vacunas, también tienden a creer información errónea sobre otros temas de salud (por ejemplo, sobre los tratamientos oncológicos o la COVID-19). No se ha abordado esta cuestión de manera explícita en ningún estudio de investigación, pero una respuesta a esta pregunta podría brindar información sobre la aplicabilidad de los hallazgos respecto a un área temática en el contenido de otras áreas temáticas. Esta información ayudaría a racionalizar el desarrollo y la puesta a prueba de las intervenciones. Por ejemplo, si supiéramos que cierto tipo de personas cree la información errónea sobre salud, política y ciencia, podríamos aplicar las intervenciones diseñadas para un campo a otros campos con mayor confianza.

## Abordar la susceptibilidad

Hay una clara necesidad de moderación de contenidos en los medios sociales, pero también necesitamos intervenciones que puedan aplicarse a gran escala para alcanzar e influir de manera efectiva en aquellas personas susceptibles de creer y transmitir información errónea sobre salud. Las intervenciones podrían estar dirigidas a mejorar la competencia digital o a concientizar acerca de la información errónea en línea. Con este fin, podría diseñarse una campaña de salud pública selectiva, pero lo primero que necesita cualquier campaña es una excelente comprensión de su público destinatario: quiénes son, qué motiva sus creencias y comportamientos, y qué puede persuadirlos con mayor probabilidad. Para comprender a nuestro público destinatario y comunicar un mensaje efectivo, necesitamos determinar las características de aquellas personas particularmente susceptibles a la información errónea. Si logramos definir quién es susceptible a la información errónea, podremos entender por qué son susceptibles. Comprender la susceptibilidad a la información errónea de esta manera podría ayudarnos a avanzar hacia su abordaje a través de intervenciones específicas de salud pública.

## Declaración.

Las opiniones expresadas en este manuscrito son responsabilidad del autor y no reflejan necesariamente los criterios ni la política de la *RPSP/PAJPH* y/o de la OPS.
